# Possibilistic Clustering-Promoting Semi-Supervised Learning for EEG-Based Emotion Recognition

**DOI:** 10.3389/fnins.2021.690044

**Published:** 2021-06-23

**Authors:** Yufang Dan, Jianwen Tao, Jianjing Fu, Di Zhou

**Affiliations:** ^1^Institute of Artificial Intelligence Application, Ningbo Polytechnic, Ningbo, China; ^2^School of Media Engineering, Communication University of Zhejiang, Hangzhou, China; ^3^Dazhou Industrial Technological Institute of Intelligent Manufacturing, Sichuan University of Arts and Science, Dazhou, China

**Keywords:** semi-supervised classification, membership function, electroencephalogram, emotion recognition, fuzzy entropy

## Abstract

The purpose of the latest brain computer interface is to perform accurate emotion recognition through the customization of their recognizers to each subject. In the field of machine learning, graph-based semi-supervised learning (GSSL) has attracted more and more attention due to its intuitive and good learning performance for emotion recognition. However, the existing GSSL methods are sensitive or not robust enough to noise or outlier electroencephalogram (EEG)-based data since each individual subject may present noise or outlier EEG patterns in the same scenario. To address the problem, in this paper, we invent a Possibilistic Clustering-Promoting semi-supervised learning method for EEG-based Emotion Recognition. Specifically, it constrains each instance to have the same label membership value with its local weighted mean to improve the reliability of the recognition method. In addition, a regularization term about fuzzy entropy is introduced into the objective function, and the generalization ability of membership function is enhanced by increasing the amount of sample discrimination information, which improves the robustness of the method to noise and the outlier. A large number of experimental results on the three real datasets (i.e., DEAP, SEED, and SEED-IV) show that the proposed method improves the reliability and robustness of the EEG-based emotion recognition.

## Introduction

Emotion is embodied by human beings: we are born with an innate understanding of emotion (Dolan, 2002; Zhang et al., 2016, 2019b). The complexity of emotion leads to different people’s understanding of emotion. Therefore, it is more difficult for machines to accurately understand emotion. As one of the hottest research topics in the field of affective computing, emotion recognition has received extensive attention from the field of pattern recognition and brain neural research (Kim et al., 2013; Mühl et al., 2014; Chu et al., 2017). In this work, we focus on emotional speculation through changes in the body. Basically, the representative internal changes of the body include blood pressure, magneto encephalogram, electroencephalogram (EEG), heart rate, respiratory rate (Mühl et al., 2014), and so on. The EEG-based traditional emotion recognition system usually has two parts: feature extraction and recognizer training (Lan et al., 2019; Zhang et al., 2020b). Jenke et al. (2014) made a comprehensive review on EEG feature extraction methods. In order to solve the recognition problem, many EEG-based emotion recognition methods have been provided recently (Musha et al., 1997; Kim et al., 2013). An ideal emotion-based brain–computer interface (BCI) can detect the emotional state through spontaneous EEG signals without explicit input from the user (Zhang et al., 2019b) and make a corresponding response to different emotional state. This kind of BCI may enhance the consumer experience in the time of an interactive session. Therefore, different approaches (Zhang et al., 2016, 2017) have been designed to recognize various emotion signals from brain waves. The latest affective BCIs (aBCI) have taken machine learning algorithms and depend on a few features with discriminative information (Jenke et al., 2014; Mühl et al., 2014). A representation of how aBCI exemplification operates is described here. When recording EEG signals, in order to generate a desired target emotion signal, it is necessary to provide users with affective stimulation of specific emotions. In the training/calibration session, the required features and corresponding emotion labels are extracted from EEG signals to train the classifier. In an ongoing BCI session, the feature extractor receives the real-time EEG data, sending the extracted features to the classifier for real-time affection classification. In this paradigm (Mühl et al., 2014), many researchers have reported a pleasing classification performance.

While effective machine learning and deep learning require a large amount of labeled data, sufficient labeled data are often difficult to obtain in real applications. Although manually labeled instances can make up for the lack of labeled instance to a certain extent, this process is time-consuming and laborious. Then, the semi-supervised learning (SSL; Zhou et al., 2003, 2014; Zhu et al., 2003; Chapelle et al., 2006; Zhu, 2008; Zhu and Goldberg, 2009; Gao et al., 2010; Zhou and Li, 2010; Zhao and Zhou, 2018; Tao et al., 2019; Wang Q.-W. et al., 2019; Wang T.-Z. et al., 2019; Zhang et al., 2019c) technique was proposed, which learns a model from a small amount of labeled instances and a large amount of unlabeled instances and solves the problem of insufficient labeled instance (i.e., poor generalization of the model obtained by supervised learning and inaccurate models obtained by unsupervised learning). Tu and Sun (2013) proposed a semi-supervised feature extraction method for EEG classification. Wu and Deng (2018) and Tao et al. (2015, 2016, 2017) proposed a semi-supervised classification framework based on collaborative training and differential evolution to improve the impact of random initial values of input layer parameters of neural networks on classification. Zu et al. (2019) explored to invent a semi-supervised classification method for large-scale remote sensing images based on low-rank block maps, and the reseults have been used to effectively improve classification performance, as graph-based semi-supervised learning (GSSL; Li and Zhou, 2011; Liu et al., 2012; Wang et al., 2012), with its intuitiveness and good learning performance, has been extensively studied. GSSL has two different types of inference, namely, transductive inference (Zhou et al., 2003; Zhu et al., 2003; Wang and Zhang, 2008; Wang et al., 2017) and inductive inference (Belkin et al., 2006; Gao et al., 2010; Nie et al., 2010). The transductive inference assumes that the unlabeled data in the learning process is exactly the test data, and it does not have a good prediction effect on the out-of-sample, for example, LGC (Zhou et al., 2003), GFHF (Zhu et al., 2003), LNP (Wang and Zhang, 2008), and ACA-S3VM (Wang et al., 2017), etc. The inductive inference puts all the instances together in the assumption learning process to find their commonalities and then gets a model. The important point to note is that the test instance does not exist in the training dataset. The Manifold Regularization (MR; Belkin et al., 2006) is a very common inductive GSSL inference, such as, GLSSVM (Gao et al., 2010), FME/U (Nie et al., 2010), etc., and the FME/U was proposed by Nie et al. (2010) generalized the MR framework induction.

Generally, GSSL inference requires certain assumptions. While the GSSL inference models have been employed on EEG datasets due to its effectiveness and intuitiveness, limited effort has been made on improving its performance by the clustering assumption. One of the most common assumptions is the clustering hypothesis: “Similar instances should share the same class label” (Chapelle et al., 2006; Zhu and Goldberg, 2009; Xue et al., 2011; Zhou et al., 2014; Wang Q.-W. et al., 2019; Wang T.-Z. et al., 2019). The assumption has an implicit assumption each instance should clearly belong to a certain class. We call this kind of classification hard classification. However, in real emotion recognition applications, it is difficult to strictly employ this assumption. For example, the same emotion will be understood as different emotion recognition by different subject at different/same scenario.

In order to solve the hard classification problem based on the traditional clustering assumption, Wang et al. (2012) and Zhang et al. (2019a) proposed a new semi-supervised classification method based on modified cluster assumption (SSCCM), which is a soft classification method based on clustering assumption. It constrained the similar instances that share the same label membership. Each instance could belong to multiple class labels and have corresponding membership values, which made good use of the fuzzy clustering assumption (Krishnapuram and Keller, 1993). However, its constraint condition made the total membership of each instance for different labels be 1, which may cause the label membership of some noises to be the same as the label membership of some normal instance, even for one or more classes. The label membership value of the noise may be greater than the normal instance, that is, the correlation is greater, which will cause misrecognition due to its constraint.

Toward the problem of the SSCCM method, we further develop a Possibilistic Clustering Promoting semi supervised learning for EEG-based Emotion Recognition (PCP-ER). The main idea of the method is threefold. First, each instance and its local weighted mean LWM (Bottou and Vapnik, 1992; Atkeson et al., 1997; Xue and Chen, 2007) share the similar memberships. Then, the recognition results obtained by the decision function and membership function are used to verify each other for enhancing the reliability of semi-supervised classification learning method. Finally, a regularization term about fuzzy entropy is added to increase the amount of sample discrimination information. We then obtain a membership function with stronger generalization ability, thereby overcoming the interference of noise and outlier on the recognition result, and further improving the robustness of the recognition method. In sum, the main contributions of this paper as follows:

(1) A possibilistic clustering promoting semi supervised learning for EEG-based emotion recognition (called as PCP-ER shortly) is proposed;
(2) This method introduces a regularization term about fuzzy entropy to obtain a label membership function with more generalization to overcome the influence of noise and outlier and improve the robustness of the method;
(3) A serial of experiments performed on real-world EEG datasets (i.e., DEAP, SEED, and SEED-IV) to verify the robust effectiveness and recognition reliability of the proposed framework.

The rest of paper is organized as follows. We design our framework PCP-ER in section “Proposed Framework” followed by its corresponding optimal algorithm in section “Optimization.” The Algorithm is explained in section “Algorithm description.” Section “Discussion” gives algorithm analysis including the reliability, convergence and generalization error bound. Experimental results and analysis on three real-world EEG datasets (i.e., DEAP, SEED, and SEED-IV) are reported in section “Experiment.” Finally, we draw a conclusion in section “Conclusion.”

## Proposed Framework

### Problem Statement

**FIGURE 1:**
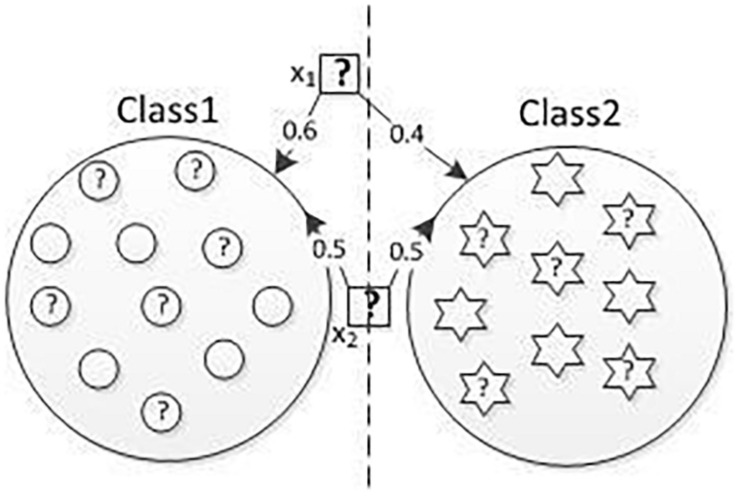
Problem description.

In real classification applications, there are some examples of EEG-based semi-supervised clustering methods where it is difficult to assign an instance explicitly belongs to only one class, such as those boundary instances. Since the hard clustering assumption implicitly constrained each instance that has a clear label assignment, the distribution of real data cannot fully be reflected, and the distribution of these boundary instances may be changed. Therefore, when a semi-supervised classification method adopts this assumption, the predictions on those boundary instances are not good. Wang et al. (2012) and Zhang et al. (2020a) proposed the classification method with modified clustering assumption to a certain extent improved the performance of the classification method based on the hard clustering assumption. Each instance will have a label membership value of a different class rather than only belonging to one class. It can reduce the “misleading” classification impact of those boundary instances. Figure 1 gives an example, in which the data in both Class1 and Class2 with a question mark are unlabeled data, the rest of other instances are labeled data, and the dashed line is the middle dividing line of the two classes. *x1* can be regarded as a boundary point or an outlier point. *x2* is certainly more like an instance of Class1 and class2 than *x1*. However, following the SSCCM, the instance is closer to one class, the membership value about this class is larger and *vice versa*. Therefore, the membership values of *x2* belonging to class1 and class2 are 0.5 and 0.5, respectively. The membership values of instance *x1* belonging to class1 and class2 are 0.6 and 0.4, respectively. The membership values of *x1* belonging to class1, which is larger than that of *x2*, making *x1* more likely to be a normal instance and *x2* an outlier. The mainly reason is the constraint term that the sum of membership from different classes of a single instance is always 1 in SSCCM, even if it is a boundary point or an outlier such as *x1*.

In order to overcome the influence of noise and outlier data on classifiers, we propose a PCP-ER.

### Formulation

In order to ensure the classification method of clustering assumption has better classification reliability and robustness, the PCP-ER method achieves the following three goals: (1) any instance should have similar label membership to its corresponding LWM; (2) the decision function and the membership function can mutually verify the classification results of a test instance and have convergence; and (3) we should try to overcome the influence caused by noise and outlier. The classification method proposed in this paper will calculate the LWM of each instance by Euclidean distance and will then obtain the decision function and label membership function through the objective function based on the square loss function with an alternating iterative strategy and utilize the fuzzy entropy to overcome the influence of noise and outlier, thereby improving the robustness of the method. Finally, an optimized classifier model with double verification is constructed by a decision function *f (x)* and a membership function *w(x)*.

Let dataset *X* = {*x*_1_, x_2_, …, x*i*, x_*i* + 1_, …, x_*n*_}, where $X_{l}={\left\{x_{i}\right\}}_{i=1}^{l}$ is the labeled data with the corresponding labels *Y*_*l*_ = {*y*_1_, *y*_2_, …, *y*_*l*_}^*T*^ ∈ ℝ^*l*×*M*^, and *n* is the total number of instances, *l*≪*n*. $X_{u}={\left\{x_{j}\right\}}_{j=l+1}^{n}$ is the unlabeled data, where *x*_*i*_ ∈ *R^d^* is the *i*−th instance with *d* dimensions. The LWM of each *x*_*i*_ (i.e., $\hat{x}$) is defined as

(1) $${\hat{x}}_{i}=\frac{\sum_{x_{j}\in Ne\left(x_{i}\right)}W_{ij}x_{j}}{\sum_{x_{j}\in Ne\left(x_{i}\right)}W_{ij}},$$

Here, *Ne*(*x*_*i*_) is composed by *k* nearest neighbors of *x*_*i*_, each of them is measured by the Euclidean Distance. *G* = (*X*, *W*) denotes a undirected weight graph, where *W* ∈ ℝ^*n*×*n*^ is a weight matrix, *W*_*ji*_ = *W*_*ij*_ ≥ 0, and the element of *W* is measured by

$$W_{ij}=\left\{ \begin{array}{cc} \exp\left(-\gamma {||x_{i}-x_{j}||}^{2}\right), & x_{i}\mathrm{is}\mathrm{the}\mathrm{nearest}\mathrm{neighbor}\mathrm{of}x_{j}\\ 0 & \mathrm{otherwise} \end{array}\right.,$$

where γ controls the local scope of the Gaussian kernel function. The larger γ is, the smaller the local scope (i.e., the width) is and *vice versa*. When γ is fixed, *W*_*ij*_ decreases monotonically with the increase in the distance between *x*_*i*_ and *x*_*j*_. Therefore, the clustering problem is transformed into a graph problem. ${\hat{X}}_{l}={\left\{\hat{x}\right\}}_{i=1}^{l}$ and ${\hat{X}}_{u}={\left\{\hat{x}\right\}}_{i=l+1}^{n}$ are the LWM of *l* labeled data and (*n* − *l*) unlabeled data, respectively. ${\left\{c_{m}\right\}}_{m=1}^{M}$ is the coded representation of *M*classes. If *x*_*i*_ belongs to the *m*−th class, then *y*_*i*_ = *c*_*m*_, the label and the category encoding are encoded according to one of the *M* categories, that is, the both of the label and the category coding is a vector of dimension *M* so PCP-ER can be directly applied to multi-class classification tasks. Let *y*_*i*_ ∈ ℝ^1×*M*^ and *c*_*m*_ ∈ ℝ^1×*M*^. If *x*_*i*_ belongs to the *m*−th class, then the *m*−th element of *y*_*i*_ is designated as 1, that is, *y*_*im*_ = 1, *m* = 1, 2, …, *M*, and the other elements of *y*_*i*_ are 0. *y*_*io*_ = 0, *o* = 1, 2, …, *M*, and *o* ≠ *m*; and the *m*−th element of *c*_*m*_ is set to 1, i.e., *c*_*mm*_ = 1, *m* = 1, 2, …, *M*. The rest of the elements in *c*_*m*_ are 0, that is, *c*_*mo*_ = 0, *o* = 1, 2, …, *M*, and *o* ≠ *m*. Except for the decision function *f*(*x*), this method also needs to define a membership function *w*(*x*), *w*(*x*_*i*_) ∈ ℝ^*M*^ for any instance *x*_*i*_, and *w*_*m*_(*x*_*i*_) is the membership of *x*_*i*_ belonging to the *m*−th class. Finally, through the improved classification method, each instance is constrained to share the same membership vector with its corresponding LWM according to the local learning principle (Bottou and Vapnik, 1992; Atkeson et al., 1997). The optimization problem of PCP-ER is formulated as

(2) $$\begin{array}{cc} \min_{f,w_{m}\left(x_{i}\right)} & \sum_{m=1}^{M}\sum_{i=1}^{n}w_{m}{\left(x_{i}\right)}^{b}{||f\left(x_{i}\right)-c_{m}||}^{2}\\ & +\lambda_{s}\sum_{m=1}^{M}\sum_{i=1}^{n}w_{m}{\left(x_{i}\right)}^{b}{||f\left({\hat{x}}_{i}\right)-c_{m}||}^{2}+\lambda {||f||}_{ℋ}^{2}\\ & -C\sum_{m=1}^{M}\sum_{i=1}^{n}\left(-w_{m}{\left(x_{i}\right)}^{b}\ln w_{m}{\left(x_{i}\right)}^{b}+w_{m}{\left(x_{i}\right)}^{b}\right)\\ s.t. & 0\leq w_{m}\left(x_{i}\right)\leq 1,m=1,\ldots ,M \end{array},$$

where λ_*s*_, λ, and *C* are regularization parameters corresponding to each term in the objective function, and the parameter *b* is an exponent on label membership. *b* is used to control the uncertainty of instances belonging to multiple classes. Specifically, when *b=1*, the value of each label membership *w*_*m*_(*x*_*i*_) is taken from {0, 1}, which will cause PCP-ER to degenerate to the original clustering classification. That is, each instance belongs to only one class. When *b* = ∞, the label membership of all instances on all classes will be equal. In order to avoid the occurrence of trivial solutions, given *b* = 2 in the subsequent derivation process of this paper, the detailed proof process of the value of *b* has been given in Krishnapuram and Keller (1993). The first term of the objective function in Eq. (2) describes the minimization of the loss by a squared loss function; the second term describes the consistency between the predictions of each instance and its corresponding LWM by adjusting the parameter λ_*s*_; the third term is a regularization term, which is used to prevent the model from over-fitting, and the complexity of the model is controlled by adjusting the parameter λ. The last term describes how to adjust the influence of noise on the model through the fuzzy entropy (Kosko, 1986; Krishnapuram and Keller, 1996), and $\sum_{m=1}^{M}\sum_{i=1}^{n}\left(-w_{m}{\left(x_{i}\right)}^{b}\ln w_{m}{\left(x_{i}\right)}^{b}+w_{m}{\left(x_{i}\right)}^{b}\right)$ calculates the fuzzy entropy. The larger the fuzzy entropy, the greater the amount of discriminative information of the sample. The model has better generalization ability. $-C\sum_{m=1}^{M}\sum_{i=1}^{n}\left(-w_{m}{\left(x_{i}\right)}^{b}\ln w_{m}{\left(x_{i}\right)}^{b}+w_{m}{\left(x_{i}\right)}^{b}\right)$ is a monotonically decreasing function about *w*_*m*_(*x*_*i*_), and it needs to adjust the balance parameter C and force *w*_*m*_(*x*_*i*_) to be as large as possible for avoiding trivial solutions. In addition, it can also make noise data have smaller different labels membership. Therefore, fuzzy entropy controls noise and outlier, making the method more robust, and its Robustness has been analyzed and proved in detail in (Krishnapuram and Keller, 1996).

For labeled instances, the label membership function is defined as

(3) $$w_{m}\left(x_{i}\right)=\left\{ \begin{array}{cc} 1, & \mathrm{if}x_{i}\in X_{m,}\begin{array}{c} i=1,2,\ldots l,\\ m=1,2,\ldots M, \end{array}\\ 0, & \text{else} \end{array}\right.$$

where *X*_*m*_ is a subset with instances belonging to the *m*−th class. The Eq. (2) can be rewritten as

(4) $$\begin{array}{cc} \min_{f,w_{m}\left(x_{j}\right)} & \sum_{i=1}^{l}{||f\left(x_{i}\right)-y_{i}||}^{2}+\lambda_{s}\sum_{i=1}^{l}{||f\left({\hat{x}}_{i}\right)-y_{i}||}^{2}\\ & +\sum_{m=1}^{M}\sum_{j=l+1}^{n}w_{m}{\left(x_{j}\right)}^{2}{||f\left(x_{j}\right)-c_{m}||}^{2}\\ & +\lambda_{s}\sum_{m=1}^{M}\sum_{j=l+1}^{n}w_{k}{\left(x_{j}\right)}^{2}{||f\left({\hat{x}}_{j}\right)-c_{m}||}^{2}+\lambda {||f||}_{ℋ}^{2}\\ & +C\sum_{m=1}^{M}\sum_{j=l+1}^{n}\left(w_{m}{\left(x_{j}\right)}^{2}\ln w_{m}{\left(x_{j}\right)}^{2}-w_{m}{\left(x_{j}\right)}^{2}\right)\\ s.t. & 0\leq w_{m}\left(x_{j}\right)\leq 1,m=1,\ldots ,M,j=l+1,\ldots ,n \end{array}.$$

According to PCP-ER, each instance has a membership vector about all classes and each instance, and its corresponding LWM share the same membership vector.

It should be noted that, in Eq. (2), we adopt a square loss function. However, other classification loss functions can also be used to develop different semi-supervised classification methods based on the possibility clustering assumption. Compared with the Eq. (3) in SSCCM (Wang et al., 2012), the Eq. (2) relaxes the constraint that the sum of the label membership on all classes is 1, and it employs the fuzzy entropy to overcome the influence of noise and outlier for obtaining the more robust model.

## Optimization

The optimization problem of PCP-ER is a non-convex problem with regard to *f*(*x*). We adopt an alternating iterative strategy to achieve the optimization of the decision function *f*(*x*) and the label membership function *w*(*x*) in this paper, and each iteration has a closed-form solution.

Fixed *w*(*x*) firstly to optimize *f*(*x*). Since the sixth term in Eq. (4) has no calculation for *f*(*x*), we can get

(5) $$\begin{array}{cc} \min_{f} & \sum_{i=1}^{l}{||f\left(x_{i}\right)-y_{i}||}^{2}+\lambda_{s}\sum_{i=1}^{l}{||f\left(\hat{x_{i}}\right)-y_{i}||}^{2}\\ & +\sum_{m=1}^{M}\sum_{j=l+1}^{n}w_{m}{\left(x_{j}\right)}^{2}{||f\left(x_{j}\right)-c_{m}||}^{2}\\ & +\lambda_{s}\sum_{m=1}^{M}\sum_{j=l+1}^{n}w_{m}{\left(x_{j}\right)}^{2}{||f\left(\hat{x_{j}}\right)-c_{m}||}^{2}+\lambda {||f||}_{H}^{2}\\ s.t. & 0\leq w_{m}{\left(x_{j}\right)}^{2}\leq 1,m=1,\ldots ,M,j=l+1,\ldots ,n \end{array}.$$

According to the Representer Theorem, the minimization problem of Eq. (5) exists in the Reproducing kernel Hilbert space (RKHS), and its solution form can be written as $f\left(x\right)=\sum_{i=1}^{n}a_{i}K\left(x_{i},x\right)$ (Belkin et al., 2006). The minimization problem is simplified to optimize the finite-dimensional space of coefficient α_*i*_. In this paper, Mercer kernel function *K* = (*K*_*ij*_) ∈ ℝ^(*l* + *u*)×(*l* + *u*)^, *K*_*ij*_ = *K*(*x*_*i*_, *x*_*j*_), *K*_*i*_ ∈ ℝ^(*l* + *u*)×*l*^, *K*_*u*_ ∈ ℝ^(*l* + *u*)×*u*^, $K=\left[K_{l}K_{u}\right]=\left[\begin{array}{cc} K_{ll} & K_{lu}\\ K_{lu}^{T} & K_{uu} \end{array}\right],\bar{K}=\left[{\bar{K}}_{l}{\bar{K}}_{u}\right]=\left[\begin{array}{cc} {\bar{K}}_{ll} & {\bar{K}}_{lu}\\ {\bar{K}}_{lu}^{T} & {\bar{K}}_{uu} \end{array}\right]$.

***Theorem 1.*** The best solution of the original optimization problem of the Eq. (5) is $f\left(x\right)=\sum_{i=1}^{n}a_{i}K\left(x_{i},x\right)$, where

$$\begin{array}{c} \alpha =\left(Y_{l}K_{l}^{T}+\hat{V}LJ^{T}K_{u}^{T}+\lambda_{s}Y_{l}{\bar{K}}_{l}^{T}+\lambda_{s}\hat{V}LJ^{T}{\bar{K}}_{u}^{T}\right)\\ \;(K_{l}K_{l}^{T}+\hat{V}K_{u}JJ^{T}K_{u}^{T}+\lambda_{s}{\bar{K}}_{l}{\bar{K}}_{l}^{T}+\lambda_{s}\hat{V}{\bar{K}}_{u}JJ^{T}{\bar{K}}_{u}^{T}+\lambda K{)}^{-1} \end{array}$$

α_*i*_ ∈ ℝ^*M*×1^, α = [α_1_, α_2_, …, α_*n*_] ∈ ℝ^*M*×*n*^ is Lagrange multiplier matrix. *Y*_*l*_ = (*y*_1_, *y*_2_, …, *y*_*l*_) ∈ ℝ^*M*×*l*^, *y*_*i*_ ∈ ℝ^*M*×1^, *i* = 1, 2, …, *l*, $J=\left[\underset{M}{\underbrace{I_{u},\ldots ,I_{u}}}\right]\in {\mathbb{R}}^{u\times \left(M\times u\right)}$, *I*_*u*_ ∈ ℝ^*u*×*u*^ is an identity matrix, *L* = [*L*_1_, …, *L*_*M*_] ∈ ℝ^*M*×(*M*×*u*)^, *L*_*m*_ ∈ ℝ^*M*×*u*^ is a matrix with all-one vector in the *m*−th row, the other being all-zero vectors. Let *V* = [*v*(*x*_1_)…*v*(*x*_*u*_)] ∈ ℝ^*M*×*u*^, *v*(*x*_*i*_) ∈ ℝ^*M*×1^ refers to the membership values of each unlabeled instance on the *M* classes. $\hat{V}$ is a diagonal matrix with each element on the diagonal correspond to the squared values of the elements in the corresponding row in the matrix *V*.

**Proof.** Like the Eq. (1), each LWM of *x*_*i*_ in the kernel space can be rewritten as. Then *K*_*lu*_ = ⟨ϕ(*X*_*l*_), ϕ(*X*_*u*_)⟩_ℋ_, *K*_*ll*_ = ⟨ϕ(*X*_*l*_), ϕ(*X*_*l*_)⟩_ℋ_, and *K*_*uu*_ = ⟨ϕ(*X*_*u*_),ϕ(*X*_*u*_)⟩_ℋ_, where ${\bar{K}}_{ll}={\left\langle \phi \left(X_{l}\right),\hat{\phi \left(X_{l}\right)}\right\rangle }_{ℋ}$, ${\bar{K}}_{lu}={\left\langle \phi \left(X_{l}\right),\hat{\phi \left(X_{u}\right)}\right\rangle }_{ℋ}$, ${\bar{K}}_{ul}={\left\langle \phi \left(X_{u}\right),\hat{\phi \left(X_{l}\right)}\right\rangle }_{ℋ}$, and ${\bar{K}}_{uu}={\left\langle \phi \left(X_{u}\right),\hat{\phi \left(X_{u}\right)}\right\rangle }_{ℋ}$, each element ${\bar{K}}_{ij}$ can be formulated as

(6) $$\begin{array}{ccc} {\bar{K}}_{ij} & = & {\left\langle \phi \left(x_{i}\right),\hat{\phi \left(x_{j}\right)}\right)}_{ℋ}={\left\langle \phi \left(x_{i}\right),\frac{\sum_{x_{s}\in Ne\left(x_{j}\right)}W_{sj}\phi \left(x_{s}\right)}{\sum_{x_{s}\in Ne\left(x_{j}\right)}W_{sj}}\right\rangle }_{ℋ}\\ & = & \frac{\sum_{x_{s}\in Ne\left(x_{j}\right)}W_{sj}{\left\langle \phi \left(x_{i}\right),\phi \left(x_{s}\right)\right\rangle }_{ℋ}}{\sum_{x_{s}\in Ne\left(x_{j}\right)}W_{sj}}\\ & = & \frac{\sum_{x_{s}\in Ne\left(x_{j}\right)}W_{sj}K_{is}}{\sum_{x_{s}\in Ne\left(x_{j}\right)}W_{sj}} \end{array}.$$

By the *F*-norm with ${||X||}_{F}^{2}=tr\left(X^{T}X\right)$, all matrices and $f\left(x\right)=\sum_{i=1}^{n}a_{i}K\left(x_{i},x\right)$ can be substituted into Eq. (5), we have

(7) $$\begin{array}{c} \;\min_{\alpha }F_{1}=tr\left(\left(\alpha K_{l}-Y_{l}\right){\left(\alpha K_{l}-Y_{l}\right)}^{T}\right)\\ \;+\lambda_{s}tr\left(\left(\alpha {\bar{K}}_{l}-Y_{l}\right){\left(\alpha {\bar{K}}_{l}-Y_{l}\right)}^{T}\right)\\ \;+tr\left({\left(\alpha K_{u}\text{J-L}\right)}^{T}\hat{V}\left(\alpha K_{u}\text{J-L}\right)\right)\\ \;+\lambda_{s}tr\left({\left(\alpha {\bar{K}}_{u}\text{J-L}\right)}^{T}\hat{V}\left(\alpha {\bar{K}}_{u}\text{J-L}\right)\right)+\lambda tr\left(\alpha K\alpha^{T}\right) \end{array}$$

Set the derivative of *F*_*1*_ w.r.t. α to zero, we have

(8) $$\begin{array}{c} \partial F_{1}/\partial \alpha =\left(\alpha K_{l}-Y_{l}\right)K_{l}^{T}+\hat{V}\left(\alpha K_{u}J-L\right)J^{T}K_{u}^{T}\\ \;+\lambda_{s}\left(\alpha {\bar{K}}_{l}-Y\right){\bar{K}}_{l}^{T}+\lambda_{s}\hat{V}\left(\alpha {\bar{K}}_{u}J-L\right)\\ \;J^{T}{\bar{K}}_{u}^{T}+\lambda \alpha K=0. \end{array}$$

According to the Eq. (8), we can get the solution of α, Theorem 1 is proved.

By Fixing *f*(*x*) to optimize *w*(*x*) from Eq. (4), we can obtain

(9) $$\begin{array}{ccc} \min_{w_{m}\left(x_{j}\right)}F_{2}=\sum_{m=1}^{M}\sum_{j=l+1}^{n}w_{m}{\left(x_{j}\right)}^{2}{||f\left(x_{j}\right)-c_{m}||}^{2} & & \\ \;+\lambda_{s}\sum_{m=1}^{M}\sum_{j=l+1}^{n}w_{m}{\left(x_{j}\right)}^{2}{||f\left({\hat{x}}_{j}\right)-c_{m}||}^{2} & & \\ \;+C\sum_{m=1}^{M}\sum_{j=l+1}^{n}\left(w_{m}{\left(x_{j}\right)}^{2}\ln w_{m}{\left(x_{j}\right)}^{2}-w_{m}{\left(x_{j}\right)}^{2}\right) & & \\ s.t.\;0\leq w_{m}\left(x_{j}\right)\leq 1,m=1,\ldots ,M, & & \\ \;j=l+1,\ldots ,n & & \end{array}.$$

***Theorem 2***. The optimal solution of the original optimization problem of the Eq. (4) is

(10) $$w_{m}\left(x\right)=\exp\left(\frac{-\left({||f\left(x\right)-c_{m}||}^{2}+{||f\left(\hat{x}\right)-c_{m}||}^{2}\right)}{2C}\right).$$

**Proof**. Set the derivative of *F*_*2*_ w.r.t. *w*_*m*_(*x*_j_) to zero, we have

(11) $$\begin{array}{ccc} \partial F_{2}/\partial w_{m}\left(x_{j}\right) & = & 2w_{m}\left(x_{j}\right){||f\left(x_{j}\right)-c_{m}||}^{2}\\ & & +2\lambda_{s}w_{m}\left(x_{j}\right){||f\left({\hat{x}}_{j}\right)-c_{m}||}^{2}\\ & & +C[2\left(w_{m}\left(x_{j}\right)\log w_{m}{\left(x_{j}\right)}^{2}\right]=0 \end{array}.$$

The solution of *w*_*m*_(*x*_*j*_) is

(12) $$w_{m}\left(x_{j}\right)=\exp\left(\frac{-\left({||f\left(x_{j}\right)-c_{m}||}^{2}+{||f\left({\hat{x}}_{j}\right)-c_{m}||}^{2}\right)}{2C}\right).$$

Therefore, the label membership vector of any instance *x* can be derived from Eq. (10), and Theorem 2 is proved.

## Algorithm Description

The optimization of PCP-ER adopts an alternating iterative strategy. PCP-ER belongs to the category of semi-supervised large boundary methods that directly seek large boundary separators. In fact, iterative learning processes are often used in various semi-supervised learning methods. The initial value of membership of an unlabeled instance can be obtained through several strategies, such as randomization strategies, fuzzy clustering techniques (such as FCM), or all zeros simply being set. Actually PCP-ER is start with labeled data to initialize the decision function *f*(*x*) in this paper. When |*F*(α_*m*_, *w*_*m*_(*x*))−*F*(α_*m*−1_, *w*_*m*−1_(*x*))| < ε*F*(α_*m*−1_, *w*_*m*−1_(*x*)), the iteration terminates, *F*(α_*m*_, *w*_*m*_(*x*)) is the value of the objective function at the *m*−th iteration, and ε is a iterative termination parameter.


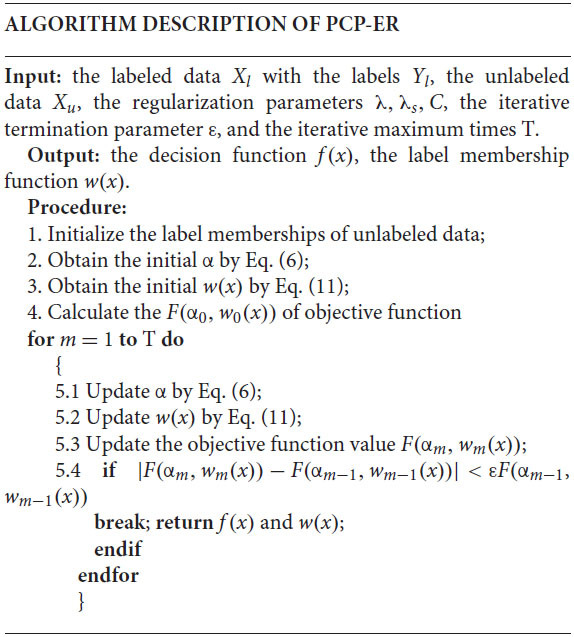


## Discussion

### PCP-ER Reliability

It takes the decision function and label membership function to identify each other’s predicted classification results in order to further enhance the reliability of PCP-ER. This leads to Theorem 3.

***Theorem 3.*** The decision function and label membership function are adopted in PCP-ER to obtain predictions, and their predictions are usually consistent (actually consistent or indirectly consistent). If the two predictions are not consistent, the predict instance may be located near the decision boundary and these predictions may be unreliable.

**Proof.** Each instance can be predicted by the decision function $y^{*}=\underset{m=1,\ldots ,M}{\arg\max}f_{m}\left(x\right)$ from Theorem 1 or the label membership function $y^{*}=\underset{m=1,\ldots ,M}{\arg\max}w_{m}\left(x\right)$ from Theorem 2. In the case of ∀*j* = 1, …, *M*;*j* ≠ *m*, if *f*(*x*) is used to predict *x* and *f*_*m*_(*x*) > *f*_*j*_(*x*), then *x* ∈ *X*_*m*_. If *w*(*x*) is taken to predict *x*, *w*_*m*_(*x*) > *w*_*j*_(*x*), the result of *x* ∈ *X*_*m*_ can also be obtained. If λ_*s*_ is fixed, *f*_*m*_(*x*) + λ_*s*_*f*_*m*_(*x*) > *f*_*j*_(*x*) + λ_*s*_*f*_*j*_(*x*) can also obtain the above consistent prediction result. When λ_*s*_ = 0, the predictions of *f*(*x*) and *w*(*x*) are consistently. When λ_*s*_ ≠ 0, *x*, and $\hat{x}$ share the same label from *f*(*x*), that is, $\underset{m=1,\ldots ,M}{\arg\max}f_{m}\left(x\right)=\underset{m=1,\ldots ,M}{\arg\max}f_{m}\left(\hat{x}\right)$, the prediction results of *f*(*x*) and *w*(*x*) are also consistent. If $f_{j}\left(\hat{x}\right)-f_{m}\left(\hat{x}\right)<\left(f_{m}\left(x\right)-f_{j}\left(x\right)\right)/\lambda_{s}$∀*j* = 1, …, *M*, *j* ≠ *m*, the prediction results of *f*(*x*) and *w*(*x*) are also consistent. If *x* is located near the decision boundary, the prediction is obviously different between *x* and $\hat{x}$, and it is possible that $\hat{x}$ and *x* are located in different class, then this prediction of *x* is unreliable.

Finally, three instances can be summarized:

(1) Intrinsic consistent instance, where instances *x* and $\hat{x}$ get the same label by *f*(*x*), then the prediction results of *f*(*x*) and *w*(*x*) on *x* are consistent;
(2) Fake-consistent instance, where *x* is not an intrinsic consistent instances, but $f_{j}\left(\hat{x}\right)-f_{m}\left(\hat{x}\right)<$(*f*_*m*_(*x*)−*f*_*j*_(*x*))/λ_*s*_ and the prediction results of *f*(*x*) and *w*(*x*) on *x* are still consistent;
(3) Inconsistent instance, where the prediction results of *f*(*x*) and *w*(*x*) on *x* are not consistently.

Thus this theorem is proved.

Actually, only one function is needed to predict new instances, and if the memberships of some instances are expected to be obtained, the label membership function is preferred. If these two functions are taken to predict instances at the same time, their prediction inconsistency is used to detect those boundary instances that are difficult to classify, and we do some special processing on them, such as manual labeling, to improve classification reliability. The prediction of these two functions can verify each other, and the reliability of semi-supervised classification can be enhanced by checking their consistency.

### PCP-ER Convergence

In order to prove the convergence of Algorithm 1, we have Theorem 4.

***Theorem 4.*** The sequence {*F*(α_*m*_, *w*_*m*_)} obtained from the above algorithm is convergent.

**Proof.** Since the objective function *F*(α, *w*) is a biconvex function on (α, *w*) (Gorski and Pfeuffer, 2007). Fixed *w*(*x*) and the objective function is a convex function on α, so the optimal α^∗^ can be calculated by minimizing *F*(α, *w*_*m*_) in Eq. (6) or optimizing Eq. (5) equivalently. Given α_*m* + 1_ = α^∗^, we know that *F*(α_*m* + 1_, *w*_*m*_) = *F*(α^∗^, *w*_*m*_) ≤ *F*(α_*m*_, *w*_*m*_). At this time, Fixed α_*m+1*_ and the objective function is a convex function on *w*. Therefore, the optimal *w* can be obtained by minimizing *F*(α_*m* + 1_, *w*_*m*_) in Eq. (10) or optimizing Eq. (9) equivalently. Given *w*_*m* + 1_ = *w*^∗^, we know that *F*(α_*m* + 1_, *w*_*m* + 1_) = *F*(α_*m* + 1_, *w*^∗^) ≤ *F*(α_*m* + 1_, *w*_*m*_), it can be inferred that*F*(α_*m* + 1_, *w*_*m* + 1_) ≤ *F*(α_*m* + 1_, *w*_*m*_) ≤ *F*(α_*m*_, *w*_*m*_),∀*m* ∈ *N*,{*F*(α_*m*_, *w*_*m*_)} is monotonically decreasing. Since the objective function is non-negative, it has a lower bound. Thus the theorem is proved.

### Generalization Error Bound of PCP-ER

In statistical learning theory, the VC dimension (Vapnik Chervonenkis dimension; Vapnik, 1995) provided a generalization error bound method that can analyze machine learning (Bishop, 2006). Therefore, this paper selects the VC dimension method to analyze the generalization error bound of PCP-ER.

***Theorem 5.*** (PCP-ER generalization error bound) Let *H* be the RKHS. The generalization error bound of the learning function *f*^Φ^ ∈ *H* that satisfies the following Eq. (13) at the probability 1 − δ(0 < δ < 1):

(13) $$\begin{array}{ccc} R\left(f^{\Phi }\right) & \leq & \varepsilon +\frac{1}{l}\sum_{i=1}^{l}L\left(f^{\phi }\left(x_{i}\right),y_{i}\right)+\\ & & \frac{1}{n-l}\sum_{m=1}^{M}\sum_{i=l+1}^{n}w_{m}^{2}\left(x_{i}\right)L\left(f^{\phi }\left(x_{i}\right),c_{m}\right)\\ & \leq & \varepsilon +\frac{1}{n}\sum_{i=1}^{n}L\left(f^{\phi }\left(x_{i}\right),y_{i}\right) \end{array},$$

where *R*(*f*^Φ^) is the expected error, it is the PCP-ER generalization error; the right side of the inequality is the upper bound of the generalization error, $\varepsilon =\sqrt{\frac{1}{2n}\left(\ln d+\ln\frac{1}{\delta }\right)}$ is a constant, *d* is the number of functions in the hypothesis space, *n* is the number of samples, and δ is the probability of a function occurrence in the hypothesis space. Since $\frac{1}{n}\sum_{i=1}^{n}L\left(f\left(x_{i}\right),y_{i}\right)$ is the empirical error of the traditional semi-supervised classification method. According to the design idea of PCP-ER, $\frac{1}{l}\sum_{i=1}^{l}L\left(f\left(x_{i}\right),y_{i}\right)$ is the empirical risk of labeled data and $\frac{1}{n-l}\sum_{m=1}^{M}\sum_{i=l+1}^{n}w_{m}^{2}\left(x_{i}\right)L\left(f\left(x_{i}\right),c_{m}\right)$ the empirical risk of unlabeled data in (14), and 0 ≤ *w*_*m*_(*x*_*i*_) ≤ 1, *m* = 1, …, *M*;*i* = *l* + 1, …, *n*. It follows that the second inequality in (13) is true. Compared with the empirical error of traditional semi-supervised classification methods, PCP-ER has a smaller generalization error bound and a better generalization.

By analysis of Theorem 5, the generalization error of this method can be adjusted and controlled by the membership function *w*_*m*_(*x*), which makes it possible to obtain better generalization.

## Experiment

This section will compare the PCP-ER method with the latest semi-supervised recognition method, the hard PCP-ER method, the PCP-ER method joint features by multiple kernel functions (called as MKPCP-ER for short), the PCP-ER method with the features from deep learning (called as DLPCP-ER for short), respectively. The emotion recognition results of all these methods are compared on the real EEG-based datasets [i.e., DEAP (Koelstra et al., 2012), SEED (Zheng and Lu, 2015), and SEED-IV (Zheng et al., 2019)]. It aims to study the following three issues:

(1) How does PCP-ER compare to the latest semi-supervised classification methods?
(2) How does PCP-ER compare to MKPCP-ER, DLPCP-ER and hard PCP-ER?
(3) How does the regularization parameterλ_*s*_affect the intrinsic consistence of PCP-ER?

### Datasets Description

There are a few existing EEG datasets that can be used for affective states investigation. In this paper, we use three publicly available datasets: DEAP (Koelstra et al., 2012), SEED (Zheng and Lu, 2015), and SEED-IV (Zheng et al., 2019).

The DEAP dataset contains 32 experimental subjects. While recording physiological signals, each subject needs to watch 40 1-min music videos as emotional stimuli. The resulting dataset includes 32-channel EEG signals, 4-channel electroencephalogram, 4-channel electromyogram, respiration, plethysmograph, Galvanic Skin Response, and body temperature. Each subject recorded 40 EEG trials, each trial corresponding to an emotion caused by a music video. After watching each video, subjects need to immediately evaluate their truly-felt emotion from five dimensions: valence (related to pleasantness level), arousal (related to excitation level), dominance (related to control power), liking (Related to preference), and familiarity (related to stimulating knowledge). The rating ranges from 1 (weakest) to 9 (strongest) except for familiarity, which is rated from 1 to 5. The EEG signals were recorded by a Biosemi Active Two device at a sampling rate of 512 Hz and down-sampled to 128 Hz.

The original SEED dataset contains 15 experimental subjects. The movie clips are intended to elicit three emotions—positive, neutral, and negative emotions—and five movie clips are assigned to each emotion. All subject need to experience three EEG recording sessions, with two consecutive recording experiments separated by 2 weeks. In each experiment, each subject watch 15 movie clips, each of which was about 4 min long, to induce the desired emotions. The same 15 movie clips were used in all three experiments. The result dataset contains 15 EEG trials for each subject in each experiment and 5 trials for each emotion. EEG signals (EEG signals) are recorded by 62-channel^1^ ESI NeuroScan equipment, with a sampling rate of 1,000 Hz and down-sampling to 200 Hz.

For the SEED-IV dataset, a total of 168 movie clips in the material pool containing four emotions (happy, sad, fear, and neutral), 44 participants (22 women, college students) were asked to evaluate their emotional state when watching the movie clips [Scoring in the two dimensions of valence and arousal; -5 ∼ 5)]. The valence scale ranges from sad to happy. Arousal is measured from calm to excited. Finally, 72 movie clips with the highest recognition are carefully selected from the material pool for the four emotion-evoking experiments. Each movie clip is about 2 min long. The experiment contains 15 experimental subjects. In order to investigate the stability of the model over time, each experimental subject needs to make a total of three experimental records in different periods, to avoid repetition, each movie clip is used for only one trial by the same subject during the three experimental periods. Each experimental for one subject contains 24 trials (each 6 trials correspond to one emotion). The resulting dataset consists of 45 experiments for all subjects. A 62-channel ESI NeuroScan System was used to record the experimenter’s EEG signal. The sampling rate was 1,000 Hz, and the sample was down-sampled to 200 Hz. SMI eye-tracking glasses records the eyes movement.

### Baselines

This experimental part compares the PCP-ER method in this paper with LapSVM (Belkin et al., 2006), LapRLS (Belkin et al., 2006), TSVM (Joachims, 1999)^2^, meanS3VM (Li et al., 2009)^3^, and SSCCM (Wang et al., 2012)—five newest semi-supervised classification methods.

LapSVM is the Laplacian Support Vector Machine. This method takes the manifold hypothesis for semi-supervised classification. The loss function is the hinge loss function. According to the Laplacian graph, it searches a maximum-face decision function the entire data distribution.

LapRLS is the Laplacian regularized least squares. The method also uses the manifold hypothesis for semi-supervised classification, but the loss function is the least square loss function.

TSVM is the Transduced Support Vector Machine. This method uses the clustering assumption, in order to find an interface on labeled and unlabeled data, so as to guide the classification boundary through low-density regions.

MeanS3VM is a type of semi-supervised SVM based on the mean value of unlabeled data. The clustering assumption is also adopted, which actually contains two implementation methods (Krishnapuram and Keller, 1996), namely the meanS3VM-iter method based on alternating optimization and the meanS3VM-mkl method based on multiple kernel learning.

Semi-supervised classification method based on modified cluster assumption is a new semi-supervised classification method based on modified clustering assumption. The clustering assumption is also used, its purpose is to find a membership function and a decision function on labeled and unlabeled data, so that similar instances should share similar label membership, and one instance can belong to multiple Classes.

Besides, we then also compare the PCP-ER method with the hard PCP-ER, which only uses the hard clustering assumption. It is supposed in hard PCP-ER that each instance clearly belongs to only one class, which can be formulated in Eq. (14), where each *c*_*i*_ ∈ ℝ^*M*^ is an one-hot vector.

(14) $$\begin{array}{ccc} \min_{f,y_{j}} & & \sum_{i=1}^{l}{||f\left(x_{i}\right)-y_{i}||}^{2}+\lambda_{s}\sum_{i=1}^{l}{||f\left({\hat{x}}_{i}\right)-y_{i}||}^{2}\\ & & +\sum_{j=l+1}^{n}{||f\left(x_{j}\right)-y_{j}||}^{2}+\lambda_{s}\sum_{j=l+1}^{n}{||f\left({\hat{x}}_{j}\right)-y_{j}||}^{2}\\ & & +\lambda {||f||}_{ℋ}^{2}\\ s.t. & & y_{j}\in \left\{c_{1},\ldots ,c_{M}\right\},j=l+1,\ldots ,n \end{array}.$$

The objective function in Eq. (14) can also be readily solved by the same strategy as adopted in our PCP-ER.

### Experimental Setting

In this section, we will give the experimental settings of the three datasets on the compared methods.

The DEAP dataset is a three binary classification (including valence, arousal, and dominance). One experimental subject contributed 40 samples. Then, the training set includes 40 × 32 = 1,240 samples from 32 experimental subject, and the test set contains 40 test samples from test subjects. In the DEAP training set, there are three settings: the first one contains 10 labeled instances, the second one contains 50 labeled instances, and the third one contains 100 labeled instances. Furthermore, each setting is related to 12 subsets of labeled data and average performance results on unlabeled data. As with other machine learning algorithms, these state-of-the-art semi-supervised classification algorithms require certain hyper-parameters to be set. For some hyper-parameters, we set them to the default values suggested by their authors. Table 1 gives details of the other hyper-parameters used in this experiment. Here, you need to use a linear kernel and an RBF kernel. When 10 labeled instances are provided, the width parameter in the RBF kernel is set to the average distance between the instances, and when there are 50 labeled instances, leave-one-subject-out cross-validation is to be taken over the labeled data. When there are 100 labeled instances, 10-fold cross-validation is used on the labeled data, and finally a better result is selected between these two kernels.

The SEED dataset is a three-category dataset, including negative, neutral, and positive. One subject contributes 45 samples. The training set includes 45 × 14 = 630 samples from 14 subjects, a total of 210 samples per session, and 45 samples from the test subject. The training data contains 10 labeled and rest unlabeled instances. The linear kernel and a leave-one-subject-out cross-validation method a taken during this process to evaluate emotion recognition accuracy. As with other machine learning algorithms, these state-of-the-arts algorithms that require certain hyper-parameters to be set. For some hyper-parameters, we set them to the default values suggested by their authors. Table 2 gives details of the hyper-parameters used in this experiment. *C* is used here to describe the divergence of the dataset. Different *C* values can be obtained according to different datasets, and $\bar{x}=\frac{\sum_{j=1}^{n}x_{j}}{n}.$

**TABLE 1:** Details of hyper-parameters on SEED dataset.

Method	Hyper-parameters
LapSVM	*C*_1_ = 1, *C*_2_ = 0.1
LapRLS	See above
TSVM	See above
MeanS3VM-iter	See above
MeanS3VM-mkl	See above
SSCCM	$\lambda =0.1,\lambda_{s}=0.1,m=5,\varepsilon =10^{-3},C=\frac{\sum_{j=1}^{n}||x_{j}-\bar{x}||}{n},$
PCP-ER	See above
Hard PCP-ER	See above

**TABLE 2:** Details of hyper-parameters on SEED-IV dataset.

Method	Hyper-parameters
LapSVM	*C*_1_ = 1, *C*_2_ = 0.1
LapRLS	See above
TSVM	See above
MeanS3VM-iter	See above
MeanS3VM-mkl	See above
SSCCM	$\lambda =0.1,\lambda_{s}=0.1,m=5,\varepsilon =10^{-3},C=\frac{\sum_{j=1}^{n}||x_{j}-\bar{x}||}{n},$
PCP-ER	See above
Hard PCP-ER	See above

The SEED-IV dataset is a four-category dataset: happy, sad, neutral, and fear. One subject contributes 72 samples. The training set includes 72 × 14 = 1,008 samples from 14 subjects; there are three sessions, a total of 336 samples per session, and 72 samples from the test subject. The training data contains 10 labeled instances, and the others are unlabeled instances. This process uses a linear kernel and a leave-one-out cross-validation method to evaluate classification accuracy. As with other machine learning algorithms, these state-of-the-arts algorithms that require certain hyper-parameters to be set. For some hyper-parameters, we set them to the default values suggested by their authors. Table 3 gives details of the hyper-parameters used in this experiment. *C* is used here to describe the divergence of the data set. Different *C* values can be obtained according to different datasets, and $\bar{x}=\frac{\sum_{j=1}^{n}x_{j}}{n}$.

**TABLE 3:** Details of hyper-parameters on DEAP dataset.

Method	Hyper-parameters
LapSVM	*C*_1_ = 100, *C*_2_ = 0.1
LapRLS	See above
TSVM	See above
MeanS3VM-iter	See above
MeanS3VM-mkl	See above
SSCCM	λ = 1,λ_*s*_ = 0.1, *m* = 5,ε = 10^−3^
Hard PCP-ER	See above
PCP-ER	See above

### Experimental Results and Analysis

Specifically, in the following tables (i.e., Tables 4, 5) of experimental results, thebold values in each column indicate the best accuracy in all these tables, and the bold values in last column from Table 5 indicate the best average performance results achieved by the compared methods. The last Avg. column shows the average performance of each method on all data sets. In Tables 4, 5, the consistency rate of PCP-ER on different settings of dataset is given in the last row. From the results of Tables 4, 5, we can draw the following conclusions.

**TABLE 4:** Performance about PCP-ER and the latest methods on DEAP dataset.

Methods	DEAP		
	10 Labels	50 Labels	100 Labels
LapSVM	49.22	53.05	63.22
LapRLS	50.06	57.49	63.46
TSVM	44.70	47.66	52.49
MeanS3VM-iter	49.83	53.43	59.17
MeanS3VM-mkl	52.11	58.47	60.54
SSCCM	**56.54**	61.31	63.33
PCP-ER	55.7	**62.40**	**66.71**
Consis. rate	0.9827	0.9943	1.00

**TABLE 5:** Performance comparison among PCP-ER and the latest methods on SEED and SEED-IV datasets.

Methods	Seed				Seed-IV			
	Session1	Session2	Session3	Avg.	Session1	Session2	Session3	Avg.
LapSVM	52.26	49.46	58.39	53.37	58.00	51.88	56.33	56.33
LapRLS	52.08	50.55	57.16	53.26	57.29	51.04	55.72	55.72
TSVM	49.83	47.29	53.44	50.19	55.73	45.20	50.60	50.6
MeanS3VM-iter	55.27	49.71	58.21	54.40	58.08	48.95	56.49	56.49
MeanS3VM-mkl	60.02	**51.20**	58.78	56.67	60.29	51.68	57.25	57.25
SSCCM	61.78	50.68	60.11	57.52	59.38	51.17	61.78	61.78
PCP-ER	**62.13**	50.04	**60.48**	**57.55**	**62.45**	**52.30**	**62.36**	**62.36**
Consis. rate	0.991	0.99	1.00	0.994	1.00	0.988	0.999	0.999

#### Results on DEAP

The experimental comparison among PCP-ER and the six latest methods on DEAP with different number of labeled samples. The experimental results are shown in Table 4. The consistency rate gradually approaches to 1 which increases as the labeled samples increase. Since the SSCCM is used to deal with normal data, it obtains the best performance with 10 labeled samples. The possible reason is that the samples are normal. However, the SSCCM method is slightly better than the PCP-ER method in this case, and the PCP-ER method has the best performance in most cases with the increase of the number of labeled samples. It shows that the clustering hypothesis with fuzzy entropy can overcome the influence of noise and outliers in semi-supervised classification.

#### Results on SEED and SEED-IV

We performed an experimental comparison among PCP-ER and the six latest methods on each session with 10 labels of SEED and SEED-IV datasets. The experimental results are shown in Table 5. The average performance of the proposed method is the best. In addition, the consistency rate is close to 1. Although the consistency rate on Session 2 of SEED-IV dataset isn’t good, its value is as high as 0.988. It is worth noting that only method MeanS3VM-mkl performs slightly better than PCP-ER method on session 2 of SEED dataset, the possible reason is that the MeanS3VM-mkl employed multiple kernels and enriched features of dataset. Nevertheless, the performance of the PCP-ER method is also closely followed, and the proposed method has the best performance of all the other datasets. It shows that the clustering hypothesis with fuzzy entropy can overcome the influence of noise in semi-supervised emotion recognition on SEED and SEED-IV.

#### Multiple-Kernel Learning

We further evaluate the effectiveness of our method with different kernel functions (called as MKPCP-ER for short) to present instances from each dataset. Given the empirical kernel mapping set ${\left\{\phi_{k}\right\}}_{k=1}^{℧}$, each mapping *X*_*a*_ into ℧ different kernel spaces, each *X* corresponds to each dataset [i.e., DEAP (Koelstra et al., 2012), SEED (Zheng and Lu, 2015), and SEED-IV (Zheng et al., 2019)], and we can integrate them orthogonally to the final space by concatenation, i.e., $\tilde{\phi }\left(x_{i}\right)={\left[\phi_{1}{\left(x_{i}\right)}^{T},\phi_{2}{\left(x_{i}\right)}^{T},\ldots ,\phi_{℧}{\left(x_{i}\right)}^{T}\right]}^{T}\in {\mathbb{R}}^{℧n_{a}}$, for *x*_*i*_ ∈ *X*_*a*_, *n*_*a*_(*a* = 1, 2, 3) training samples in each dataset. The final kernel matrix in this new space is defined as $K_{new}=\left[{\tilde{K}}_{1};{\tilde{K}}_{2};\ldots ;{\tilde{K}}_{℧}\right]$, where ${\tilde{K}}_{i}$ is the kernel matrix in the *i*−th feature space about *i*−th dataset. Therefore, besides the above mentioned Gaussian kernel, we additionally employ another three types of kernels in MKPCP-ER: Laplacian kernel $K_{ij}=\exp\left(-\sqrt{\sigma }||x_{i}-x_{j}||\right)$, inverse square distance kernel *K*_*ij*_ = 1/(1 + σ||*x*_*i*_−*x*_*j*_||^2^), and inverse distance kernel $K_{ij}=1/\left(1+\sqrt{\sigma }||x_{i}-x_{j}||\right)$.

**FIGURE 2:**
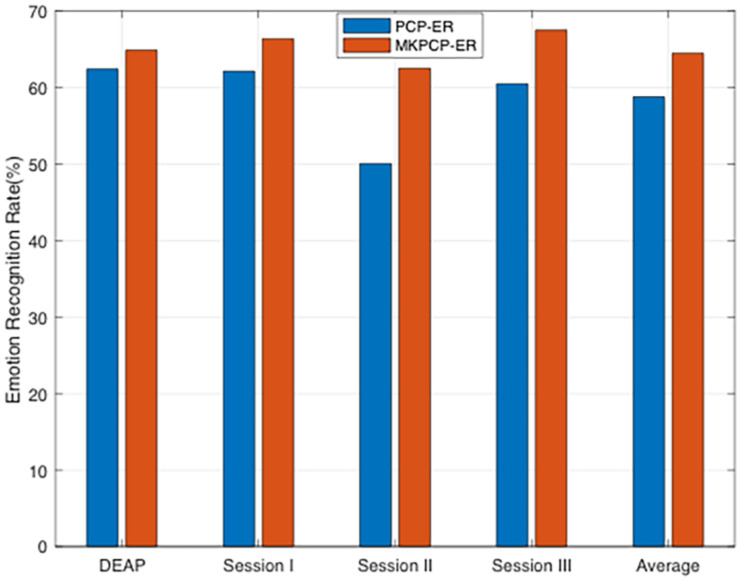
PCP-ER with multi-kernel learning.

It can be clearly seen from Figure 2 that MKPCP-ER is obviously better than PCP-ER in terms of mean accuracies in all cases, which justifies that the multi-kernel trick can improve the quality of semi-supervised emotion recognition on each dataset.

#### Deep Features Learning

In the past decades, deep learning attracts more and more attention due to its powerful representation ability and dramatic improvement over the traditional shallow methods. The EEG emotion recognition method based on deep learning has also been widely used and has achieved better recognition effect than traditional methods. For example, in Zheng and Lu (2015) proposed a deep belief network for EEG emotion classification; in Song et al. (2018) and Zhong et al. (2020), authors used graphics to model multi-channel EEG features and then classified EEG emotion on this basis; Li et al. (2018) proposed a new neural network model, which uses time information for EEG emotion recognition task. We therefore additionally compare our PCP-ER method with the recently proposed deep transfer learning models VGG16 (Simonyan and Zisserman, 2014) and ResNet50 (He et al., 2016; it is the same as Res50 in the following) for emotion recognition using deeply extracted features. In our PCP-ER, we can tackle the problem of deep emotion recognition with two steps: firstly, a higher-level feature extraction is learnt in an unsupervised fashion from all available datasets using the popular deep architectures (e.g., VGG16 or Res50); secondly, our PCP-ER is trained on the transformed data of all datasets and then used to classify test dataset. For fair comparison, however, we follow the experimental setup in Zhou et al. (2018) and Zhu et al. (2017). Specifically, we first fine-tune pre-trained deep models (e.g., VGG16, and Res50) by using the labeled samples in the dataset, and then use these fine-tuned CNN models to extract the features from EEG in dataset. Finally, we perform emotion recognition using PCP-ER on these deeply extracted features. In the context of our experiments, we denote our methods with different deep models as PCP-ER+VGG16, PCP-ER+Res50, respectively. As for VGG16 and Res50, we use their released source codes and fine-tune the pre-trained deep models, respectively.

**FIGURE 3:**
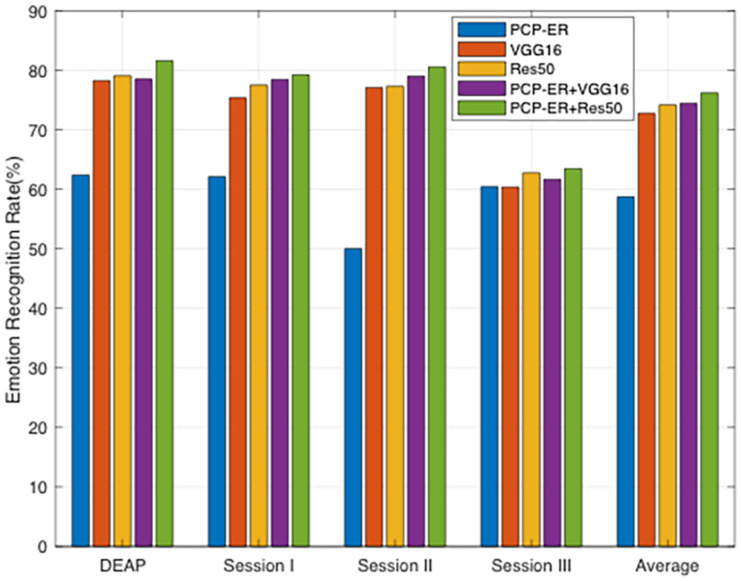
Emotion recognition accuracies (%) of different methods using deeply extracted features.

All experimental results are reported in Figure 3. As can be seen from this plot, the deep learning methods are originally proposed to learn each dataset features, while our proposed method aims to improve the anti-interference ability, namely, their methods focus on feature learning, while our work focuses on emotion recognition. So our proposed method can be used to further improve the recognition accuracies by employing the features extracted by deep models, i.e., VGG16 and Res50, rather than the original EEG patterns. This indicates that the recognition-level constraint can preserve all discriminative structures of labeled samples for the guidance of unlabeled samples recognition, which demonstrates the effectiveness of PCP-ER framework. From the plot bars of Figure 3, it can be observed that PCP-ER+VGG16 consistently outperforms VGG16, while PCP-ER+Res50 is consistently better than Res50, which demonstrates that our PCP-ER method is complementary to the two deep learning methods VGG16 and Res50 by exploiting more features from dataset.

#### Comparison Between PCP-ER and Hard PCP-ER

Tables 6, 7 show the performance results of PCP-ER and hard PCP-ER on DEAP, SEED, and SEED-IV datasets, respectively. Specifically, in the following tables (i.e., Tables 6, 7) of experimental results, the bold values in each column indicate the best accuracy, and the bold values in last column of different dataset indicate the best average performance results achieved by the compared hard PCP-ER and PCP-ER method. From the overall observation of the results in Tables 6, 7, when there are more training samples or more labeled samples, the PCP-ER and hard PCP-ER methods are better. Moreover, the performance and average performance of PCP-ER are better than hard PCP-ER. It demonstrates that the PCP-ER method based on membership degree is effective and robust on EEG-based emotion recognition.

**TABLE 6:** Performance comparison between hard PCP-ER and PCP-ER on DEAP dataset.

Methods	DEAP			
	10 Labels	50 Labels	100 Labels	Avg.
hard PCP-ER	53.27	58.44	62.69	58.1
PCP-ER	**55.7**	**62.40**	**66.71**	**61.1**

**TABLE 7:** Performance comparison between hard PCP-ER and PCP-ER on SEED and SEED-IV datasets.

Methods	SEED				SEED-IV			
	Session1	Session2	Session3	Avg.	Session1	Session2	Session3	Avg.
hard PCP-ER	60.57	47.32	56.78	54.89	60.38	49.08	61.66	57.04
PCP-ER	**62.13**	**50.04**	**60.48**	**57.55**	**62.45**	**52.30**	**62.36**	**59.03**

### Consistency Analysis

**FIGURE 4:**
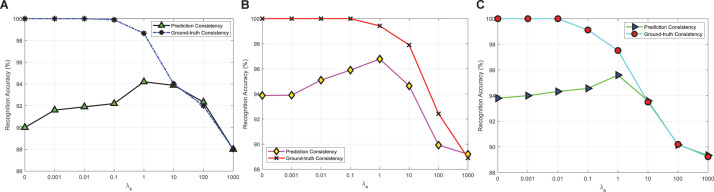
Prediction consistency and ground-truth consistency between *f*(*x*) and *w*(*x*) with different λ_*s*_ values on **(A)** DEAP; **(B)** SEED; and **(C)** SEED-IV.

Figure 4 shows the experimental results of PCP-ER actual consistency rates corresponding to different λ_*s*_ values {0, 0.001, 0.01, 0.1, 1, and 101001000} on DEAP, SEED, SEED-IV three datasets. In Figures 4A,C, when λ_*s*_ is small enough, the prediction consistency rate can reach 100%, and then the consistency rate gradually decreases with the increase of λ_*s*_. Since the indirect consistency instance becomes the inconsistent instance, it finally becomes equal to the ground-truth consistency rate. In Figure 4B, when λ_*s*_ is in the range of 1 to 1000, the prediction consistency rate and the ground-truth consistency rate are not equal, although the trend of change is the same with Figures 4A,C, and the possible reason is that there are the least training samples on SEED dataset. In addition, in Figures 4A–C, with the increase of λ_*s*_ to 1, the ground-truth consistency rate also increases. When λ_*s*_ continues to increase, the ground-truth consistency rate begins to decrease. It may be that when λ_*s*_ is far less than or greater than 1, the PCP-ER will focus more on the emotion recognition of samples or LWM samples and not on its prediction consistency.

## Conclusion

The existing GSSL methods construct undirected weight grapy, which is sensitive or not enough robust to noise or outlier from EEG patterns. At the end, we proposed PCP-ER for EEG-based affective recognition. By adding the regularization term of fuzzy entropy, the amount of discrimination information of samples is increased, and a more generalized emotion recognizer is obtained through learning to overcome the negative effects of noise and outliers and improve the robustness of the method. Experimental results on the three real datasets DEAP, SEED, and SEED-IV show that the proposed method improves more the reliability and robustness of emotion recognition than competing algorithms. Moreover, both PCP-ER with multi-kernel and depth features extraction obtained better performance than PCP-ER. These tricks can improve the quality of the PCP-ER method on each dataset. However, in the process of optimization, how to obtain an effective combined multi-kernel function or kernel space and how to analyze and demonstrate the consistency of the proposed method in theory are issues worthy of further discussion.

## Data Availability Statement

Publicly available datasets were analyzed in this study. This data can be found here: http://epileptologie-bonn.de/cms/upload/workgroup/lehnertz/eegdata.html.

## Ethics Statement

Ethical review and approval was not required for the study on human participants in accordance with the local legislation and institutional requirements. The ethics committee waived the requirement of written informed consent for participation.

## Author Contributions

All authors listed have made a substantial, direct and intellectual contribution to the work, and approved it for publication.

## Conflict of Interest

The authors declare that the research was conducted in the absence of any commercial or financial relationships that could be construed as a potential conflict of interest.

## Footnotes

1 The 62 EEG channels include AF3, AF4, C1, C2, C3, C4, C5, C6, CB1, CB2, CP1, CP2, CP3, CP4, CP5, CP6, CPZ, CZ, F1, F2, F3, F4, F5, F6, F7, F8, FC1, FC2, FC3, FC4, FC5, FC6, FCZ, FP1, FP2, FPZ, FT7, FT8, FZ, O1, O2, OZ, P1, P2, P3, P4, P5, P6, P7, P8, PO3, PO4, PO5, PO6, PO7, PO8, POZ, PZ, T7, T8, TP7, and TP8.

2 The codes of LapSVM, LapRLS and TSVM are available from https://www.csie.ntu.edu.tw/~cjlin/libsvm/

3 The code is downloaded from http://www.lamda.nju.edu.cn/CH.Data.ashx
